# Supplementary figures and images for: The bZIP transcription factor BIP1 of the rice blast fungus is essential for infection and regulates a specific set of appressorium genes
Source: PLoS Pathog. 2024 Jan 22;20(1):e1011945. doi: 10.1371/journal.ppat.1011945 (PMC10833574; doi:10.1371/journal.ppat.1011945)

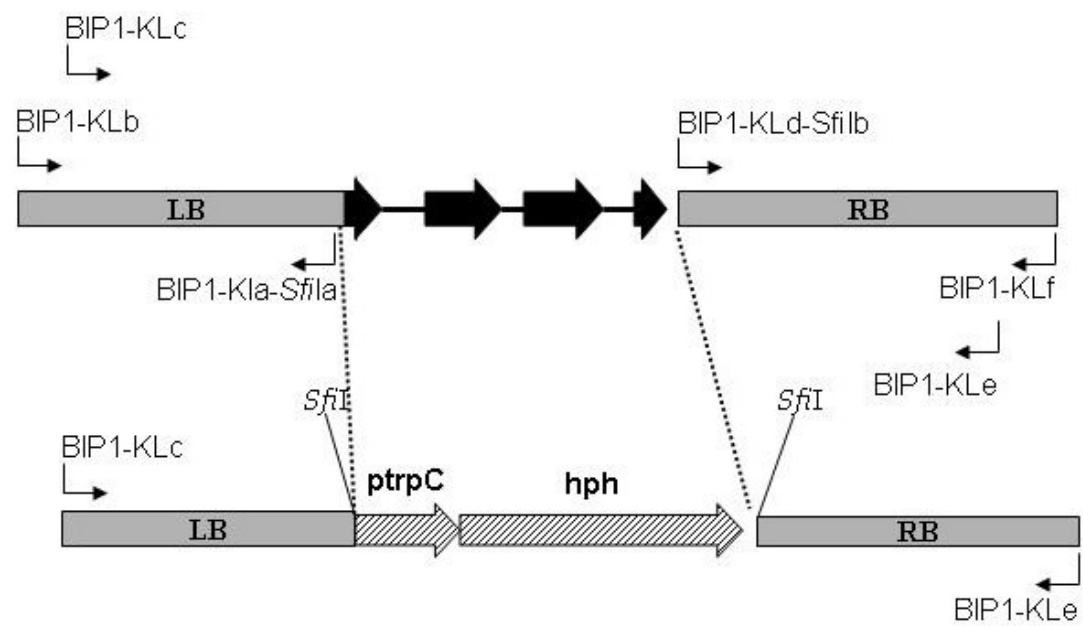

Supplement: S1 Fig — A. M. oryzae BIP1 locus regions used to construct the gene replacement vector. 1.2 kb and 1.36 kb genomic regions (respectively Left Border and Right Border grey boxes) flanking the BIP1 ORF were amplified using P1.2 genomic DNA and primers shown as arrows (S5 Table). The four exons of BIP1 are shown as black boxes separated by introns. B. Structure of the BIP1 locus in the Δbip1 mutants. Hatched boxes correspond to the hygromycin resistance cassette. Grey boxes represent the Left and Right Border sequences flanking the BIP1 ORF used to construct the gene replacement vector. C. Analysis of the transformants by Southern blot. Genomic DNA was digested with HindIII and probed with the 1.36 kb RB fragment (top) and 0.85 kb hph cassette (bottom). Lanes 1, 2, and 3, Δbip1 transformants; lane 4, Wild type (P1.2). (PDF) [file ppat.1011945.s001.pdf]

A

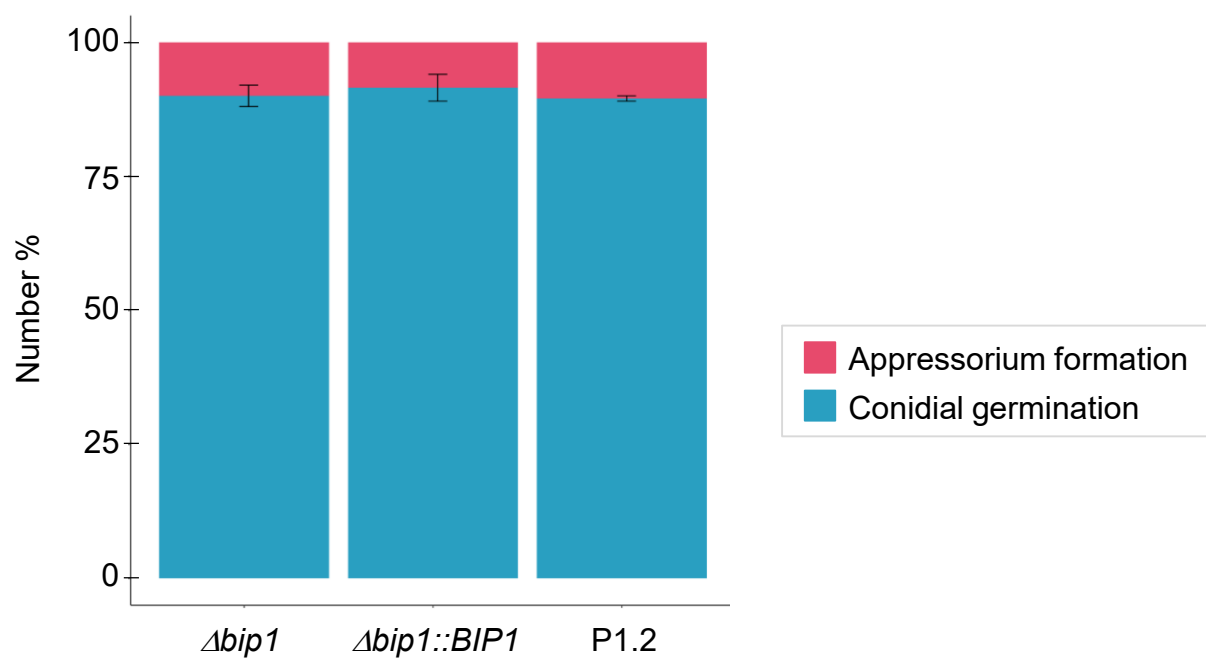

B

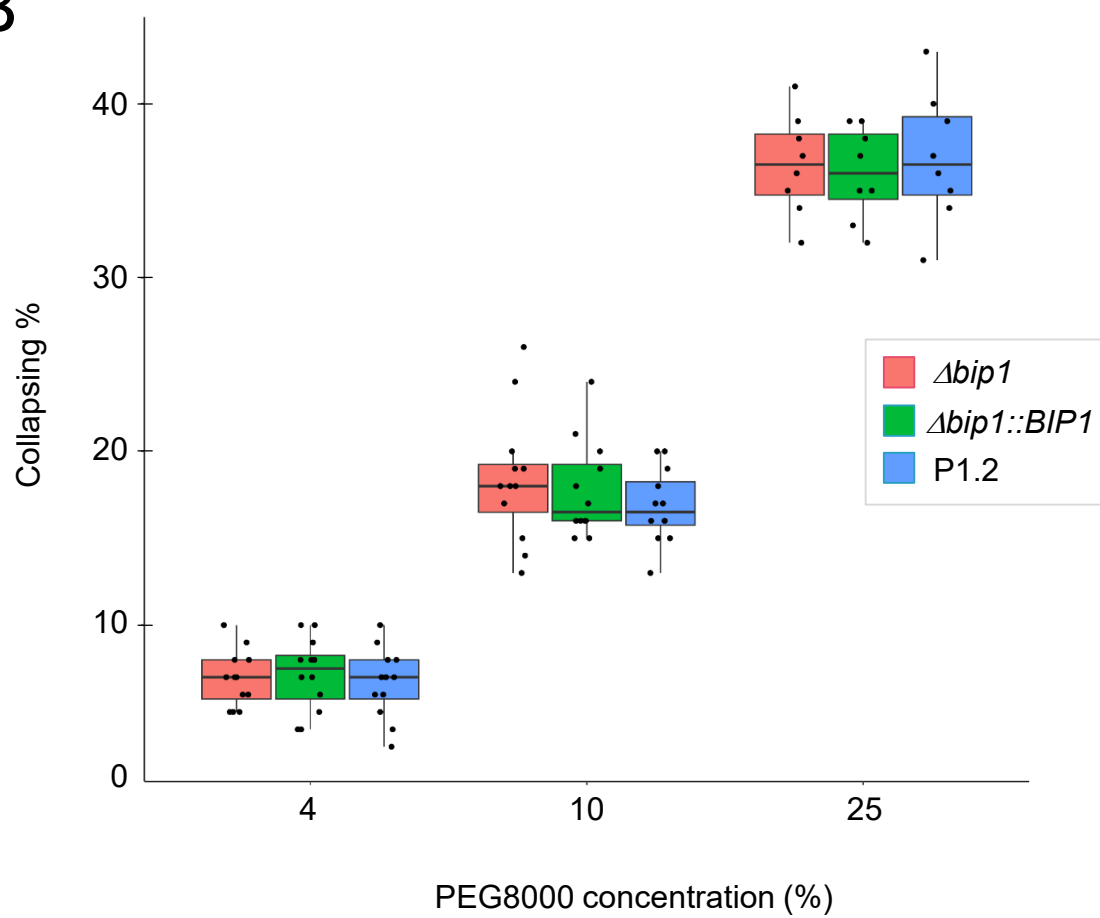

Supplement: S2 Fig — A. Differentiation of appressoria was observed at 16 hai on Teflon membrane. Error bars represent standard deviations. B. Collapsing of appressoria formed on Teflon membrane was assessed 24 hai with PEG8000 at 4%, 10% and 25%. Tree independent experiments with each three different replicate samples were performed. No significant differences in collapsing rates were observed between the three strains (Anova: F = 0.50, P = 0.74, Df = 4). (PDF) [file ppat.1011945.s002.pdf]

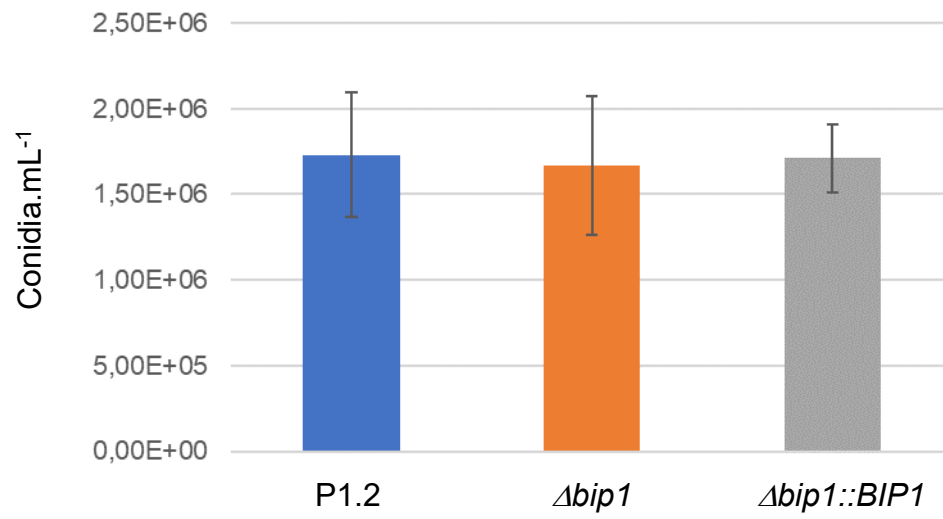

Supplement: S3 Fig — Conidiation rates (conidia.mL-1) after 12 days of rice medium cultures. No significant differences in sporulation rates were observed between Δbip1 and wild-type P1.2 (t-Test day 5, p = 0.84) or between Δbip1 and Δbip1::BIP1 complemented strain (t-Test day 5, p = 0.87). (PDF) [file ppat.1011945.s003.pdf]

A

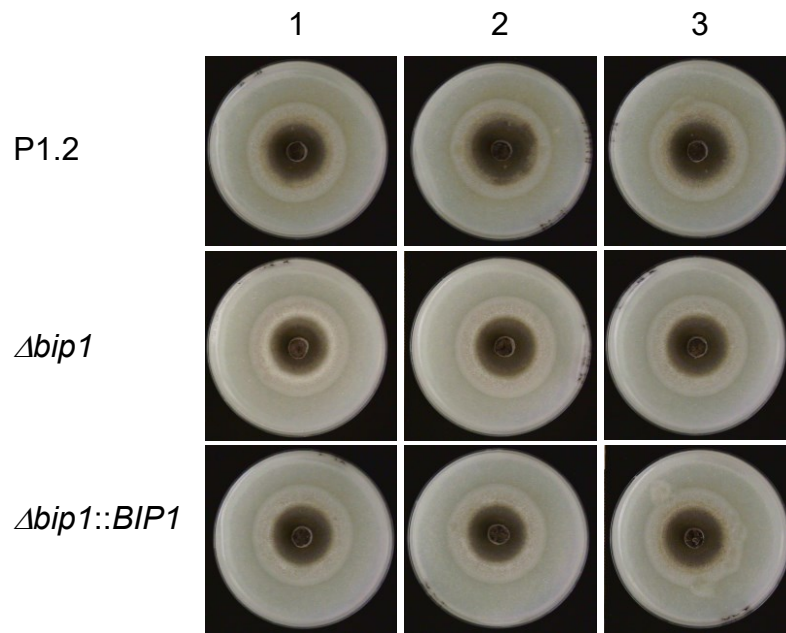

B

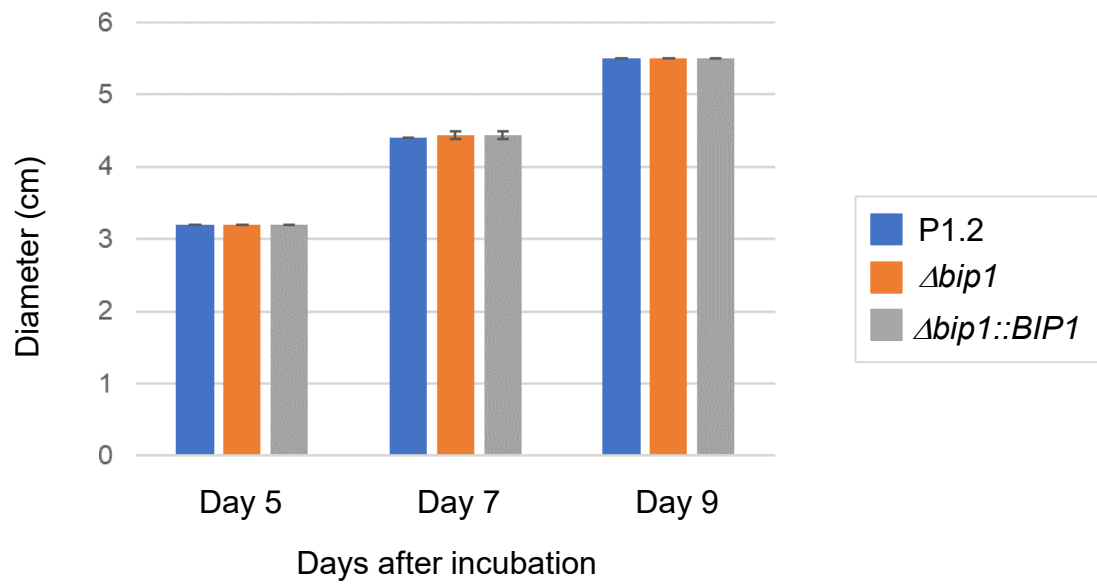

Supplement: S4 Fig — A. Five-day-old rice medium cultures of P1.2, Δbip1 and Δbip1::BIP1. B. Mycelial growth diameters (cm) of 5, 7 and 9-day-old rice medium cultures. No significant difference was observed between Δbip1 and wild-type P1.2 (t-Test day 5, p = 1) or between Δbip1 and Δbip1::BIP1 complemented strain (t-Test day5, p = 1). B shows results from three independent experiments each performed with three different replicate samples. Error bars are standard deviations. (PDF) [file ppat.1011945.s004.pdf]

**A**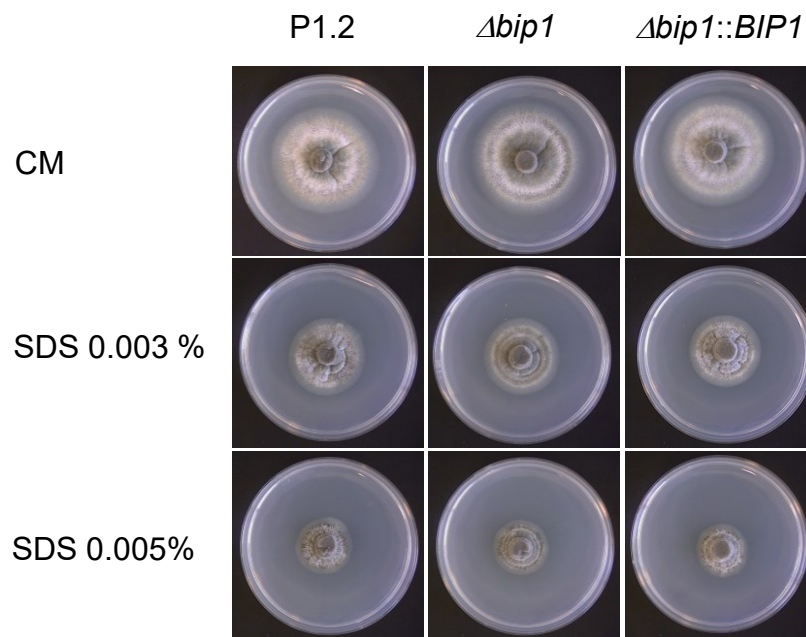**B**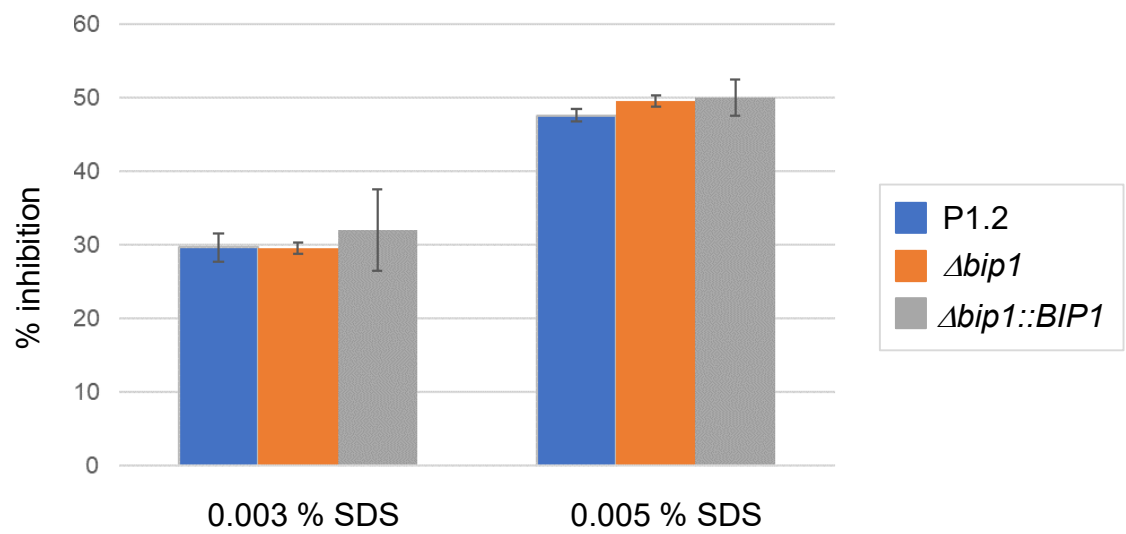

C

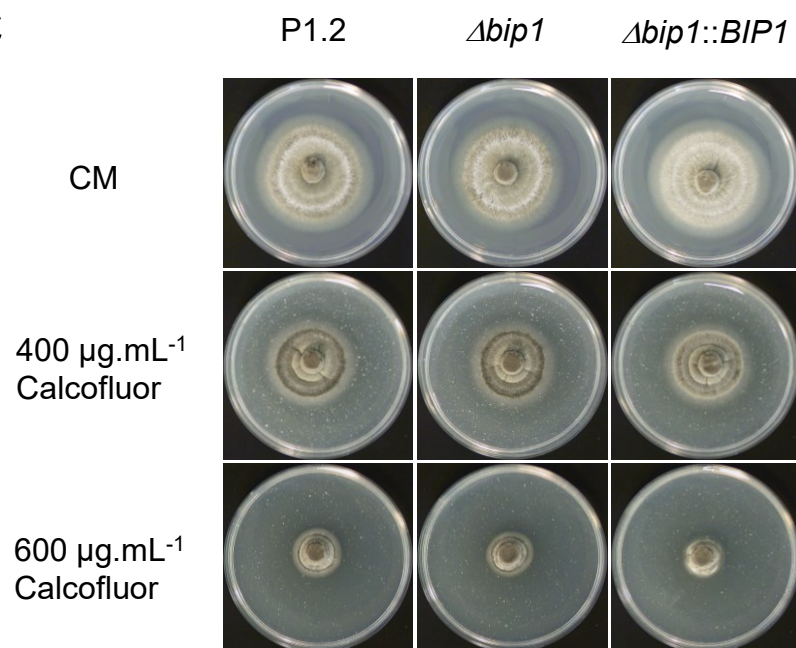

D

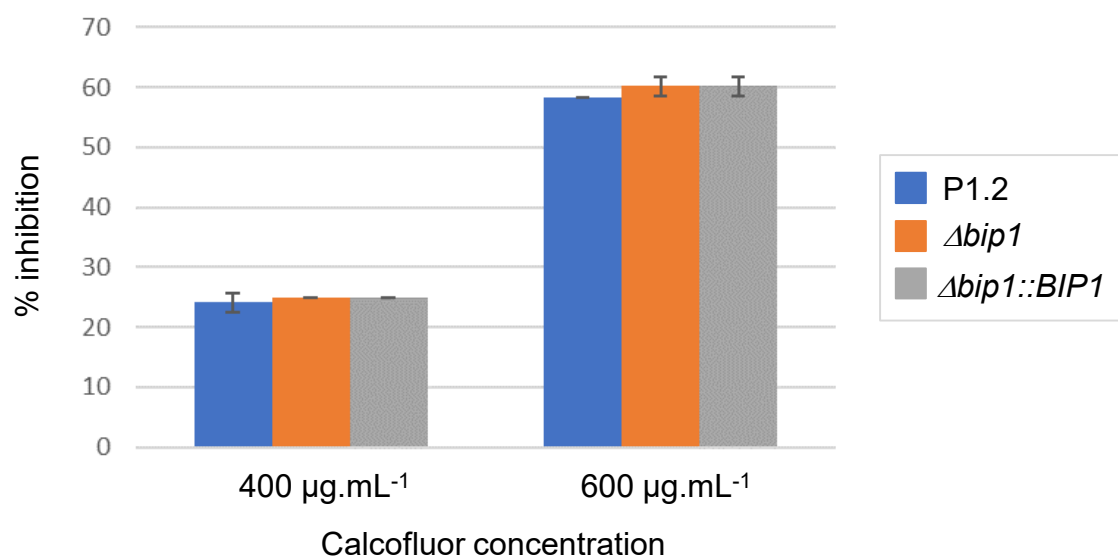

E

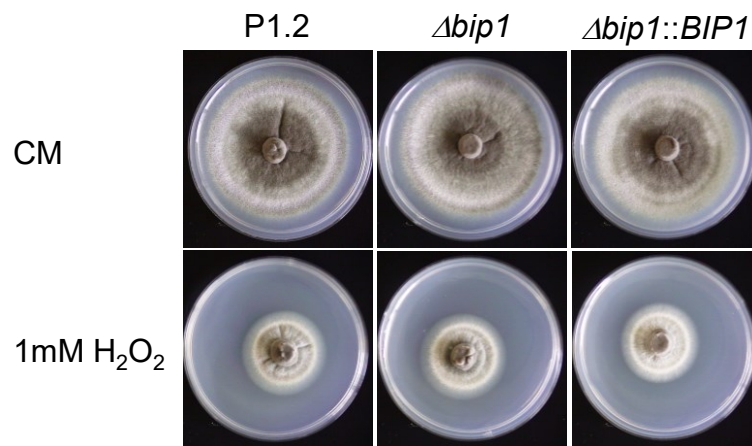

F

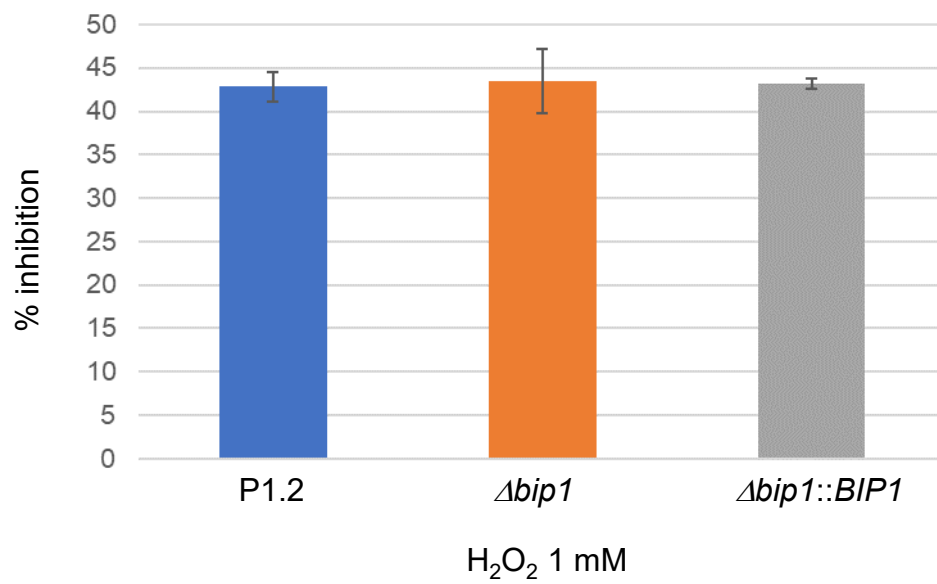

Supplement: S5 Fig — P1.2, Δbip1, Δbip1::BIP1 strains were cultured on CM medium without or with stress agent (cell wall integrity stressors: 0.003 or 0.005% SDS (A,B), 200 or 400 μg.mL-1 calcofluor (C,D); oxidative stress inducer: 1mM H2O2 (E,F). Colony diameters of strains were measured 5 days after inoculation for cell wall stress (SDS, calcofluor) and 7 days after inoculation for oxidative stress (H2O2). Growth inhibitions were calculated with following formulae (Inhibition rate = (the diameter of untreated strain—the diameter of treated strain)/(the diameter of untreated strain) X 100%). Three independent replicates with three samples per replicate were performed (B,D,F). Error bars are standard deviations. No significant difference was observed between Δbip1 and P1.2, as well as Δbip1::BIP1 (T-test p-values > 0.05). (PDF) [file ppat.1011945.s005.pdf]

**P1.2**

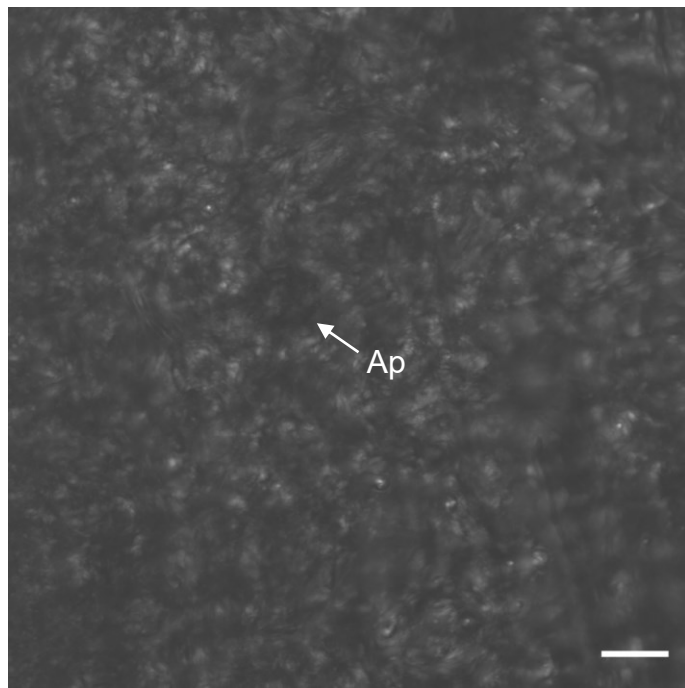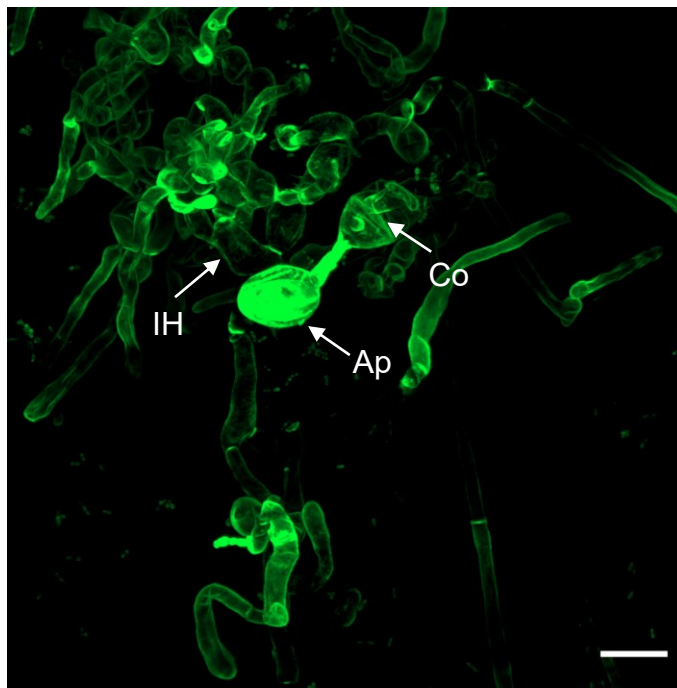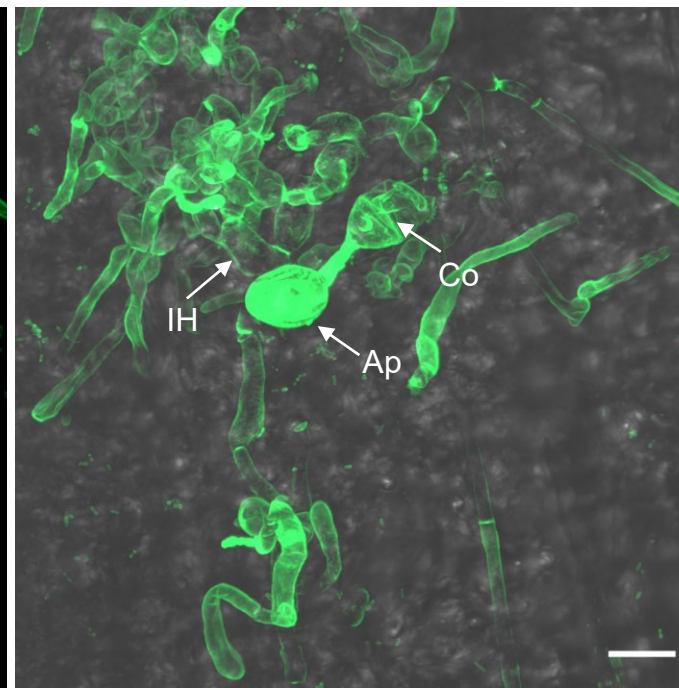

***Δbip1***

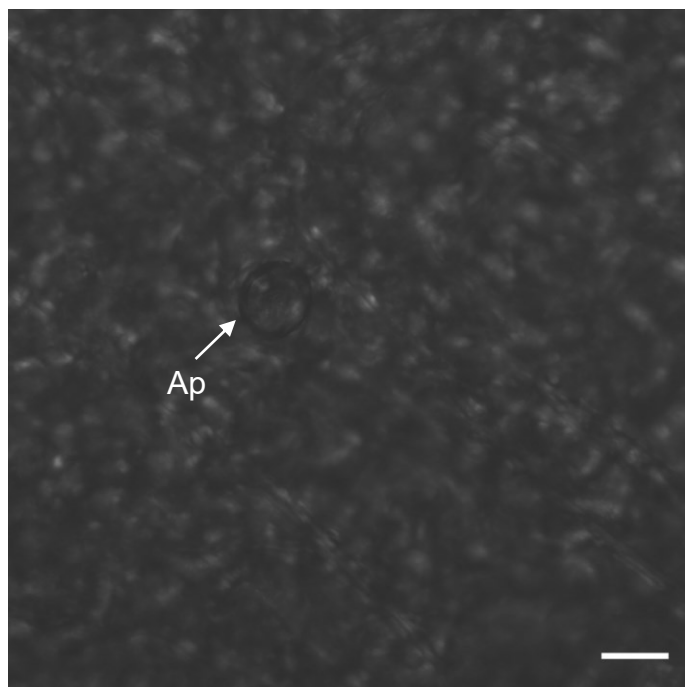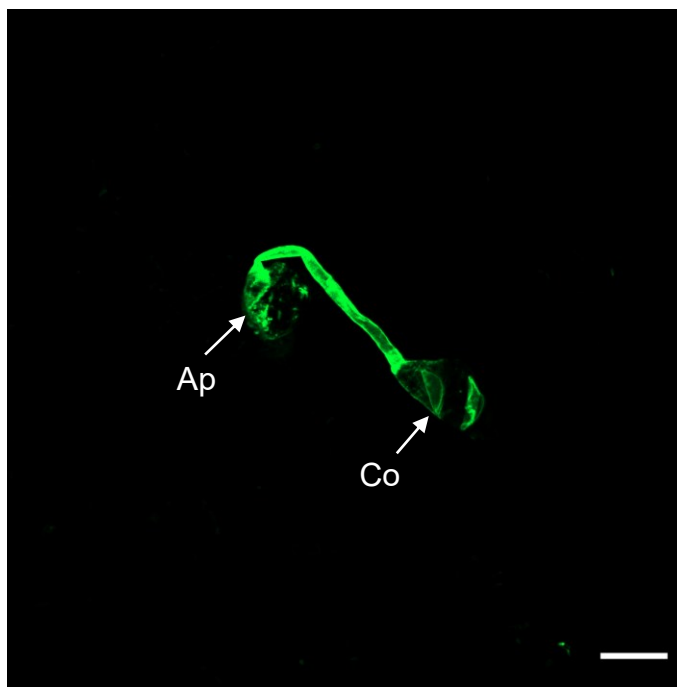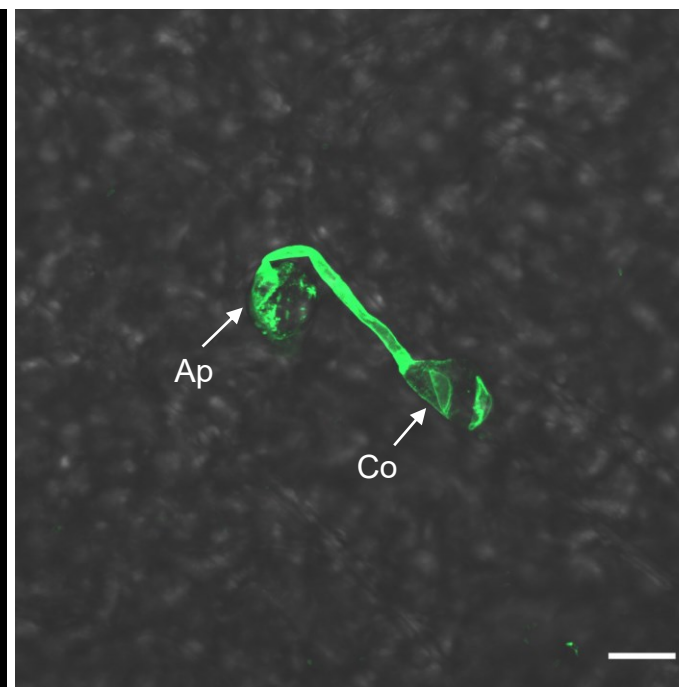

Supplement: S6 Fig — No infection hyphae were observed in epidermal cells of rice sheaths infected with Δbip1 mutant whereas epidermal cells of rice sheaths infected with P1.2 were filled with infection hyphae resulting from penetration events. Fluorescence of WGA-Alexa488-stained fungal cells was excited with 488 nm light and is shown in green. Ap: appressorium, Co: conidium, IH: invasive hyphae, size bar = 10μm. (PDF) [file ppat.1011945.s006.pdf]

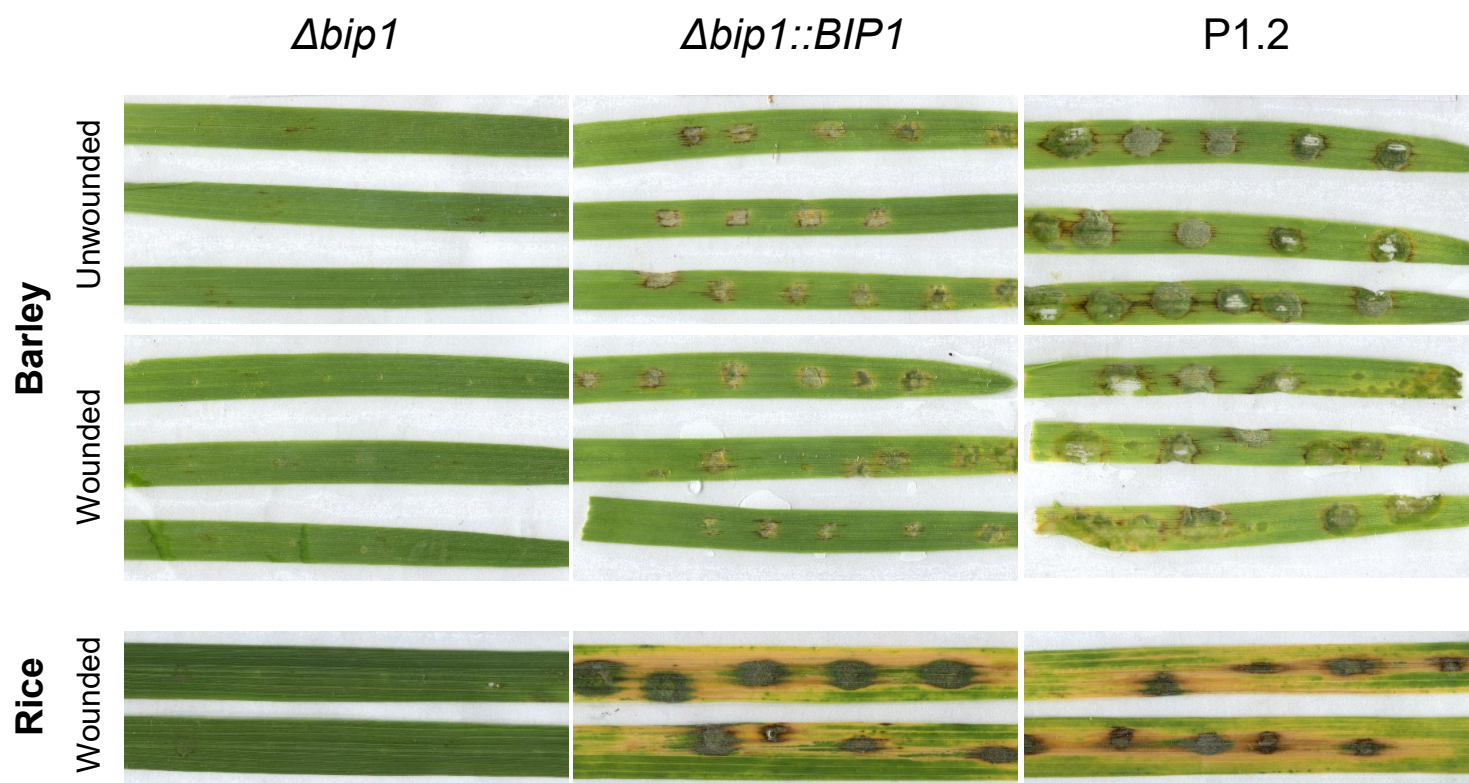

Supplement: S7 Fig — Droplets of conidial suspensions (5.104 conidia.mL-1) were deposited on detached intact or wounded barley leaves or detached wounded rice leaves. Photos were taken 5 dai. (PDF) [file ppat.1011945.s007.pdf]

A

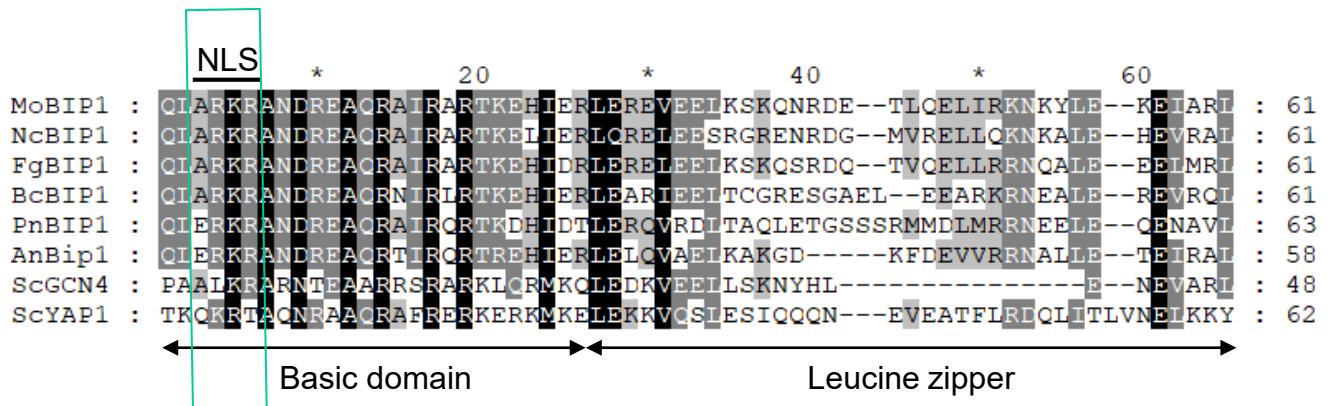

B

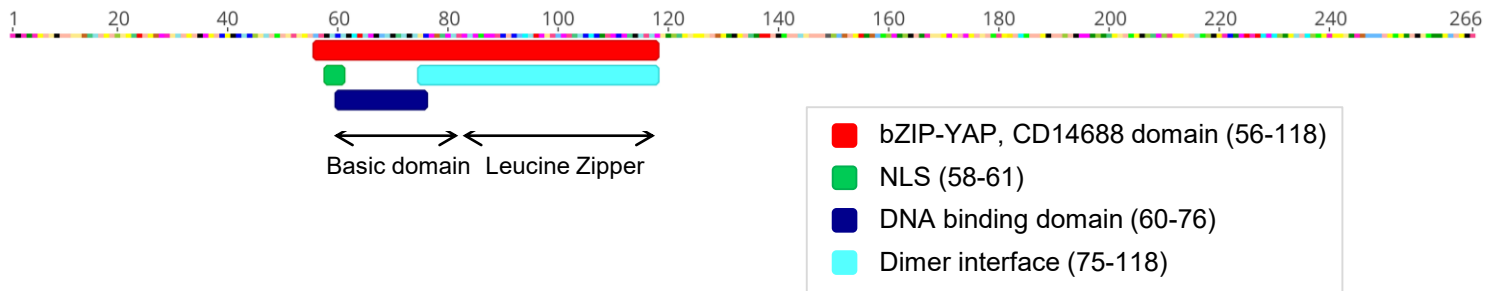

Supplement: S8 Fig — A. Alignment of bZIP domains from BIP1, BIP1 orthologues from N. crassa (NcBIP1, NCU03847), F. graminearum (FgBIP1, FGRAMPH1_01G06311), B. cinerea (BcBIP1, Bcin09g05210), A. nidulans (AnBIP1, ANIA_00825) and P. nodorum (SNOG_11592). S. cerevisiae Gcn4 (ScGCN4, YEL009C) and S. cerevisiae Yap1 (ScYAP1, YML007W) TFs were added for comparison. bZIP domains were extracted from protein sequences and aligned using Clustal omega. 100% identical amino acids are highlighted in black. 90–80% similar amino acids are highlighted in dark grey. 70–60% similar amino acids are highlighted in light grey. NLS: nuclear localization signal predicted using Hidden Markov Model for nuclear localization signal prediction. B. Functional domains identified in BIP1 protein using CDD database (bZIP-YAP, CD14688 domain) and previous analysis using YAP1 fungal TFs (A). (PDF) [file ppat.1011945.s008.pdf]

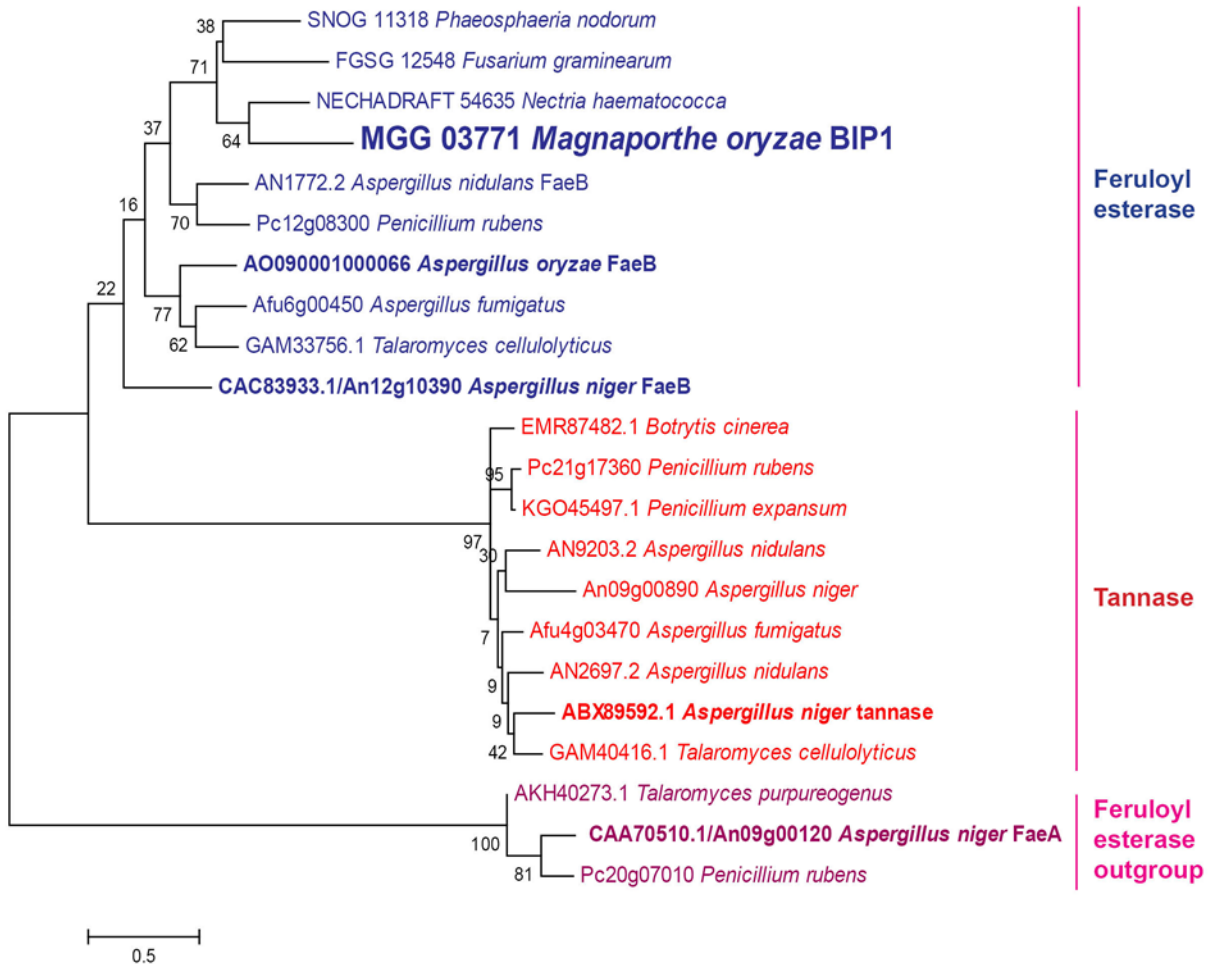

Supplement: S9 Fig — Minimal Evolution tree of selected fungal sequences encoding feruloyl esterases or tannases. A. niger FaeA and two orthologous sequences were used as an outgroup. The scale bar shows a distance equivalent to 0.5 amino acid substitutions per site. Bootstrap values (1000 bootstraps) are presented at the nodes. Biochemically characterized proteins are in bold. (PDF) [file ppat.1011945.s009.pdf]
