# Supplementary material for: The bZIP transcription factor BIP1 of the rice blast fungus is essential for infection and regulates a specific set of appressorium genes
Source: PLoS Pathog. 2024 Jan 22;20(1):e1011945. doi: 10.1371/journal.ppat.1011945 (PMC10833574; doi:10.1371/journal.ppat.1011945)
Supplement: S1 Table — (PDF) [file ppat.1011945.s010.pdf]

S1 Table. *ACE1* cluster expression in *Δbip1* during barley infection 24 hai.

Gene ID	Gene name in *ACE1* cluster	Expression in wild type P1-2 *	Expression in *ΔBIP1:hph* *	Fold change **
MGG_12447	*ACE1*	6.3x10^{-4} ± 3.7x10^{-4}	8.8x10^{-6} ± 4.4x10^{-6}	0.01
MGG_08386	*BC2*	1.3x10^{-5} ± 5.2x10^{-6}	2.4x10^{-4} ± 1.0x10^{-4}	19
MGG_08387	*CYP1*	1.2x10^{-3} ± 3.7x10^{-4}	1.1x10^{-4} ± 5.6x10^{-5}	0.09
MGG_15928	*CYP2*	1.3x10^{-4} ± 1.8x10^{-5}	1.4x10^{-3} ± 5.0x10^{-4}	11
MGG_08379	*CYP3*	1.8x10^{-4} ± 5.3x10^{-4}	3.1x10^{-6} ± 3.4x10^{-6}	0.02
MGG_08378	*CYP4*	1.4x10^{-3} ± 2.1x10^{-4}	2.4x10^{-5} ± 2.7x10^{-5}	0.02
MGG_08377	*OME1*	1.7x10^{-4} ± 1.1x10^{-4}	6.7x10^{-5} ± 4.1x10^{-5}	0.40
MGG_08381	*ORF3*	3.5x10^{-3} ± 1.1x10^{-3}	1.6x10^{-4} ± 1.6x10^{-4}	0.05
MGG_08391	*RAP1*	4.5x10^{-4} ± 1.9x10^{-4}	1.4x10^{-4} ± 1.6x10^{-4}	0.30
MGG_08380	*RAP2*	1.2x10^{-3} ± 3.7x10^{-4}	6.7x10^{-5} ± 4.1x10^{-5}	0.05

* Expression was determined by qRT-PCR. ** The ratio of expression between the *Δbip1* mutant and the wild-type control was calculated using the formula $2^{-\Delta Ct\ \Delta bip1} / 2^{-\Delta Ct\ \text{wild type}}$.
