# Supplementary material for: The bZIP transcription factor BIP1 of the rice blast fungus is essential for infection and regulates a specific set of appressorium genes
Source: PLoS Pathog. 2024 Jan 22;20(1):e1011945. doi: 10.1371/journal.ppat.1011945 (PMC10833574; doi:10.1371/journal.ppat.1011945)
Supplement: S2 Table — (PDF) [file ppat.1011945.s011.pdf]

S2 Table. Expression of 15 BIP1-regulated genes during initial stages of rice infection (16hpi).

Class	Gene	Name	Ratio *Δbip1*/P1.2 *
SSPs	*MGG_11610*	*BAS3*	0
	MGG_09693	*BAS2*	0
	MGG_00751	-	0
	MGG_12655	*AVR-Pi9*	0
	MGG_17425	-	0,002 #
	MGG_06666	-	0,016 #
	MGG_05638	-	0,15 #
	MGG_03504	-	0,22
	MGG_08428	-	0,38
	MGG_07934	-	0,45
Enzymes	*MGG_02201*	endopeptidase	0,003 #
	MGG_08480	lipase/esterase	0,13 #
	MGG_11966	cutinase	0,58
ACE1 Cluster	*MGG_12447*	*ACE1*	0
	MGG_08381	*ORF3*	0
Control genes	*MGG_05344*	snodprot 1	0,58
	MGG_03245	aldose 1-epimerase	0,76

*Expression was determined by qRT-PCR. The ratio of expression between the *Δbip1* mutant and the wild-type control was calculated using the formula $2^{-\Delta Ct\ \Delta bip1} / 2^{-\Delta Ct\ \text{wild type}}$. “0” indicates expressed in WT but not expressed in *Δbip1*. # indicates significantly different at p>0,05 according to t-test using expression values from three biological replicate samples.

Similar results were obtained in two other independant experiments.
