# Supplementary material for: The bZIP transcription factor BIP1 of the rice blast fungus is essential for infection and regulates a specific set of appressorium genes
Source: PLoS Pathog. 2024 Jan 22;20(1):e1011945. doi: 10.1371/journal.ppat.1011945 (PMC10833574; doi:10.1371/journal.ppat.1011945)
Supplement: S3 Table — (PDF) [file ppat.1011945.s012.pdf]

S3 Table. Summary of BIP1 binding to target promoter used for EMSA.

Promoter	Probe location bp	Probe sequence 5' to 3'	BIP1 Binding	Description
MGG_02201	-272 to -223	tttttttgcgtgtcaaaagaccgagtcataatgtgtttgactttcacccg	+	Peptidase
MGG_03584	-305 to -354	cacagccctccatatccctgcgtcgagaaccctgccttctcgccatacga	-	*PTH11-like*
	-462 to -413	acagccgttctcatcgactgactcgtcaaccctggattcatatactacga	+	
MGG_06535	-645 to -596	ctttgcatacacagggttcttgactcgaggtcatggtaggatttagccct	+	*PTH11-like*
MGG_08380/ MGG_08381	-208 to -255	gcaaaaggtattttcgagtcatgctcctagtcatggaataaaagatggga	+	*RAP2/ORF3*
MGG_08386	-370 to -789	aacgcctgataaccttattgatcgtttgcacttcaaactcgtttgccgac	-	*BC2 TF*
	-285 to -334	ccttccagattatcctacaacttttgccatgttgatggattcgaattatc	-	
	-197 to -246	cgatgccacaaacacaagctcaagtgcgaacgtgagccaagcattaccga	-	
	-398 to -447	cgtccaaagtttgaatttgacctgatcattagcgttctgttgaacttttt	-	
