# Supplementary material for: The bZIP transcription factor BIP1 of the rice blast fungus is essential for infection and regulates a specific set of appressorium genes
Source: PLoS Pathog. 2024 Jan 22;20(1):e1011945. doi: 10.1371/journal.ppat.1011945 (PMC10833574; doi:10.1371/journal.ppat.1011945)
Supplement: S4 Table — oryzae. (PDF) [file ppat.1011945.s013.pdf]

S4 Table. Summary of bZIP transcription factors in *M. oryzae*.

number	CD	Interpro	Super-familly	Panther	MobZIP Kong et al. 2015	MoBzip Tang et al. 2015	MoBZIP Liu et al. 2022	Locus 70-15 v8	Annotation
MoBZIP01	cd14688	IPR004827, IPR046347	SSF57959	PTHR40621	MobZIP01	MoBzip1	MoBZIP1	MGG_00342	FlbB
MoBZIP02	cd14688, cd43524	IPR004827, IPR046347	NF*	PTHR11462	MobZIP02	MoGCN4	MoBZIP2	MGG_00602	CPC1
MoBZIP03	cd21462	PR031106, IPR004827, IPR046347	SSF57959	NF	MobZIP03	Mo-Idi4	MoBZIP3	MGG_01990	IDI4
MoBZIP04	cd14687	IPR004827, IPR046347	SSF57959	PTHR19304	MobZIP04	MoBzip3	MoBZIP4	MGG_02006	bZIP
MoBZIP05	cd14688	IPR031106, IPR004827, IPR014720	SSF57959	PTHR40621	MobZIP05	MoFcr3	MoBZIP5	MGG_02632	RmsA
MoBZIP06	cd14688	IPR004827, IPR046347	SSF57959	NF	MobZIP06	MoBzip4	MoBZIP6	MGG_02865	bZIP
MoBZIP07	cd14810	IPR004827, IPR046347	SSF57959	PTHR19304	MobZIP07	MoBzip5	MoBZIP7	MGG_03288	bZIP
MoBZIP08	cd14687	IPR004827, IPR046347	SSF57959	PTHR19304	MobZIP08	MoBzip6	MoBZIP8	MGG_04009	bZIP
MoBZIP09	cd21462	NF	NF	PTHR13044	MobZIP09	NF	MoBZIP9	MGG_04122	bZIP
MoBZIP10	cd21462	IPR004827, IPR046347	SSF57959	PTHR47416	MobZIP10	MoBzip12	MoBZIP10	MGG_04758	bZIP
MoBZIP11	NF	NF	NF	NF	MobZIP11	NF	NF	MGG_05016	unknown
MoBZIP12	cd14688	IPR004827, IPR046347	SSF57959	PTHR40621	MobZIP12	MoMeaB	MoBZIP12	MGG_05306	MEAB
MoBZIP13	NF	IPR004827, IPR046347, IPR018287	SSF57959	PTHR40621	MobZIP13	MoHapX	MoBZIP13	MGG_05959	HAPX
MoBZIP14	cd21462	IPR004827, IPR046347	SSF57959	PTHR40621	MobZIP14	MoBzip10	NF	MGG_06131	bZIP
MoBZIP15	cd14688	IPR004827, IPR046347	SSF57959	PTHR40621	MobZIP15	MoBzip7	MoBZIP15	MGG_07305	bZIP
MoBZIP16	cd14688	IPR046347	SSF57959	PTHR40618	MobZIP16	MoBzip11	MoBZIP16	MGG_07925	bZIP
MoBZIP17	cd14687	IPR004827, IPR046347, IPR021755	SSF57959	PTHR19304	MobZIP17	MoAtf1	MoBZIP17	MGG_08212	AFT1
MoBZIP18	cd14710	IPR004827, IPR046347, IPR044280	SSF57959	PTHR46714	MobZIP18	MoHac1	MoBZIP18	MGG_09010	HAC1
MoBZIP19	cd14686	IPR004827, IPR046347	SSF57959	PTHR13044	MobZIP19	MoBzip8	MoBZIP19	MGG_10660	bZIP
MoBZIP20	NF	IPR004827	NF	PTHR13044	MobZIP20	MoBzip9	MoBZIP20	MGG_12560	MoEITF2
MoBZIP21	cd14688	IPR004827, IPR046347, IPR023167	SSF57959	PTHR40621	MobZIP21	MoAp1	MoBZIP21	MGG_12814	AP1
MoBZIP22	cd14705	IPR004827, IPR046347	SSF57959	PTHR13044	MobZIP22	MoMetR	MoBZIP22	MGG_14561	CYS3
MoBZIP23	cd14688	IPR004827, IPR046347	SSF57959	PTHR39607	MobZIP23	MoBzip2	NF	MGG_00587	bZIP
MoBZIP24-1	cd14688	IPR004827	NF	PTHR39607	NF	NF	MoBZIP23	MGG_04024	bZIP Y34
MoBZIP24-2	cd14688	NF	NF	PTHR39607	NF	NF	NF	MGG_04024	bZIP
MoBZIP25	cd14688	IPR004827	NF	PTHR39607	NF	NF	MoBZIP24	MGG_04024	bZIP Y34
**MoBZIP26**	cd14688	NF	NF	PTHR37012	NF	NF	NF	**MGG_08118**	**BIP1**
**MoBZIP27**	cd14688	IPR021833	NF	PTHR37012	NF	NF	NF	**MGG_08587**	**BIP2**
MoBZIP28	cd14688	IPR046347, IPR036770	SSF57959, SSF48403	PTHR24126	NF	NF	NF	MGG_14010	bZIP

*NF: domain or gene not found.
