# Supplementary material for: The bZIP transcription factor BIP1 of the rice blast fungus is essential for infection and regulates a specific set of appressorium genes
Source: PLoS Pathog. 2024 Jan 22;20(1):e1011945. doi: 10.1371/journal.ppat.1011945 (PMC10833574; doi:10.1371/journal.ppat.1011945)
Supplement: S5 Table — (PDF) [file ppat.1011945.s014.pdf]

S5 Table. Primers used in this study.

Primers	Sequence (5'/3')	Purpose
KO1	cgagaacgccctaccacccgcccac	deletion of M. oryzae BIP1
KO2-SfiIa	cacggcctgagtggccgggttccagagggagacctaatgtg	deletion of M. oryzae BIP1
KO3	cggctaactagggagacaaagacc	deletion of M. oryzae BIP1
KO4-SfiIb	gtgggccatctaggccggagatgttaaatagtgttagccgc	deletion of M. oryzae BIP1
KO5	gtccgagtttcactttcacttgccg	deletion of M. oryzae BIP1
KO6	cccaataatatgctcccagactcc	deletion of M. oryzae BIP1
RACE-1	gatgatgatgtccggctggaaactg	5'RACE of BIP1
RACE-2	cttggagggtctcgtcgcggttctg	5'RACE of BIP1
MGG_03584.7-F	tggtgctttggctttgtcct	qPCR validation
MGG_03584.7-R	tccgaggtacgtggtaagcaa	qPCR validation
MGG_06535.7-F	actgcaagccgttgagaaagg	qPCR validation
MGG_06535.7-R	atgccagaataactgccgctg	qPCR validation
MGG_08380.7-F	ggccttgcaacttattcgtttg	qPCR validation
MGG_08380.7-R	ttgatttgctggccgcat	qPCR validation
MGG_08386.7-F	aacaaggcccacagtttcagtt	qPCR validation
MGG_08386.7-R	tgcgccaggacggaat	qPCR validation
MGG_08387.7-F	cgccaggctggtacaggta	qPCR validation
MGG_08387.7-R	gcctacgcacttccagcaa	qPCR validation
MGG_02160.7-F	ttgttgacgttgatgtggcc	qPCR validation
MGG_02160.7-R	gcagcagcataaaatcgcaa	qPCR validation
MGG_02201.7-F	aaagcgccacggttatcac	qPCR validation
MGG_02201.7-R	gatgccccttggaagttga	qPCR validation
BIP1-RL5	gctagctaatacgactcactatagggagagccaccatgactcgatcaccatc	BIP1 Recombinant protein
BIP1-RL-Strep2	tttttttttttttttattttcgaactgcgggtggctccaagcgctgagagccggcgattg	BIP1 Recombinant protein
TERMMGG_08381.7NotI+	agcaaccatggcatggaatgcggccgcttatgtctagaatacatctg	*MGG_08381.7 expression reporter vector*
TERMMGG_08381.7EcoRI-	ttacctgaattcgcctgctcattcatctc	*MGG_08381.7 expression reporter vector*
