# Supplementary material for: The bZIP transcription factor BIP1 of the rice blast fungus is essential for infection and regulates a specific set of appressorium genes
Source: PLoS Pathog. 2024 Jan 22;20(1):e1011945. doi: 10.1371/journal.ppat.1011945 (PMC10833574; doi:10.1371/journal.ppat.1011945)
Supplement: Attachment — Submitted filename: 2023_08_16_BIP1_Response to editor and reviewers-Final.docx [file ppat.1011945.s016.docx]

Dear Editor,

We would like to thank you and the reviewers for the valuable comments and suggestions, which helped us to improve our manuscript. We have carefully revised the text and the figures according to the comments and have performed additional experiments to better characterize the phenotype of the *Δbip1* mutant.

Below, we provide a point by point response to your and the reviewer’s comments that we hope will allow acceptance of the manuscript for publication in PLoS Pathogen.

Yours Sincerely

Karine Lambou

**Editor’s comments:**

The reviewers suggest how the paper might be improved, but generally hit on one of two major flaws of the paper that I am also concerned about, namely that it is not shown what the physiological basis for infection impairment is and, I might also add, it is not shown what underlying gene expression changes in *Δbip1* directly result in the loss of appressorium penetration and/ or IH development. Thus, the work currently lacks critical details about Bip1 function that need to be resolved.

With regards to appressorial physiology, it must be determined if loss of infection is due to impaired turgor or other maturation defects as suggested by Reviewers 1 and 3 or whether, as Reviewer 2 indicates, penetration pegs are formed and capable of penetrating host rice cells. I might also add that the authors should check whether Δ*bip1* appressoria are defective in mucilage for adhesion. A recent study of a mutant, Δ*sps1*, which is also Δ*bip*1-like but was not included in the list on line 517, showed it produced a peg but was reduced for adhesion, which impaired turgor, as determined using cellophane assays and turgor measurements (Rocha et al. 2020; DOI: 10.1038/s41564-020-0786-x). Thus, the authors should similarly assess Δ*bip1* appressoria on cellophane, as well as measure their turgor, in order to determine if they form pegs or have altered mucilage production that would affect adhesion and turgor. Alternatively, Δ*bip1* might form normal appressoria and pegs which penetrate host cells but fail to elaborate IH. This would be harder to assess, but not impossible by using, for example, SEM to show peg penetration takes place. I agree with Reviewer 2 that rice experiments are essential throughout. Other cellular markers of appressorium formation, as suggested by Reviewer 3, might also be informative here. Editorially, therefore, we request that turgor and penetration peg formation data be added to the paper.

**Answer:** We have added data, which show that mycelial growth, sporulation, appressorium differentiation, appressorium turgor and sensitivity to stresses (cell wall, oxidative) are not impaired in the *Δbip1* mutant (Supplemental Figures 2 to 5). Since the appressorium turgor was not altered, we did not specifically address the formation of mucilage and adhesion.

Text modification, lines 148-150:

*MGG_08118* deletion mutants were also not affected in their conidiation (**S3 Fig**), mycelial growth (**S4 Fig**), or resistance to cell wall and oxidative stress (**S5 Fig**).

We also addressed the potential invasion of host leaves by the Δ*bip1* mutant by confocal microscopy. We did not detect infective hyphae in the mutant, even not very small, early infective hyphae such as those formed in the *M. oryzae* tig1 histone deacetylase mutant (Ding et al. 2010, figure 3 & 4; doi: 10.1105/tpc.110.074302) (Figure 3 B and Supplemental Figure 3). Confocal microscopy also did not show any sign for the formation of a penetration peg. Since early penetration pegs may be difficult to visualize by confocal microscopy, we remained cautious in the interpretation of this result.

Text modification, lines 151-154:

However, the ability of this mutant to penetrate into barley and rice epidermal cells was completely abolished (**Fig3**, **S6 Fig**). Indeed, confocal microscopy showed that the mutant was arrested before the development of early infectious hyphae, and, presumably, did not produce a penetration peg (**Fig3, S6 Fig**).

With regards to my second point, I am also concerned that it is not known which Bip1-controlled genes are required for appressoria penetration and/ or IH elaboration. **Knocking out in WT some downstream candidate genes to show they result in a similar Δ*bip1*-phenotype**, or constitutively expressing one or two candidates in Δ*bip1,* might be useful here.

**Answer:** Six BIP1 target genes (*ACE1, SYN2, BAS2*, *BAS3, SLP2* and *AvrPi9*) have already been knocked out individually in published studies. In none of the cases this led to a reduction in fungal virulence. We conclude from this that the individual genes regulated by BIP1 make incremental contributions to fungal virulence and/or that they are redundant among each other. To test this hypothesis, multiple genes would have to be knocked out to mimic the Δ*bip1*-phenotype. However, to our opinion, performing these experiments or mutating additional BIP1-regulated genes individually would go beyond the scope of this study that presents already a very substantial amount of new and original data.

Relevant text in the discussion, lines 396-400:

Deletion of *ACE1/SYN2* and the effectors *BAS2*, *BAS3, SLP2* or *AvrPi9* did not lead to mutants with reduced pathogenicity [36–39]. These results suggest that individually BIP1-regulated genes are not critical for infection. However, the functions they fulfill collectively seem to be crucial for appressorium-mediated penetration and invasive infectious growth

Other good points are raised by the reviewers, such as **whether conidiation is affected** in Δ*bip1*, and these should all be addressed.

**Answer:**

We have added data that show that conidiation is unchanged in the mutant (Supplemental Figure 3).

Text modification, lines 148-150:

*MGG_08118* deletion mutants were also not affected in their conidiation (**S3 Fig**), mycelial growth (**S4 Fig**), or resistance to cell wall and oxidative stress (**S5 Fig**).

**Reviewer’s comments:**

**Part I – Summary**

.

Reviewer#3:

1- Figure 5. I’m curious as to whether the use of 3xeGFP was necessary due to low or undetectable fluorescence with single copy eGFP, or whether this was simply chosen by default?

**Answer**: The fluorescence of BIP1:GFP using a single GFP was too low to be detected. We had to use a triple GFP to detect BIP1:3xeGFP fluorescence.

2- Further, was nuclear localization of Bip1-3xeGFP present in all/most nuceli, or was there heterogeneity within the population? It might be nice to have some quantitation, but this is not absolutely necessary. It might also be compelling to perform time-lapse imaging to resolve precisely when Bip1-3xeGFP becomes localized to the appressorium nucleus. How long after mitotic division and nuclear migration is it before fluorescence is detectable in the nucleus? Again, not essential, but if it can be done easily, it might add some value.

**Answer:** There was no heterogeneity in the nuclear localization of BIP1-3xGFP fluorescence in the population of appressoria observed. The fluorescence was only visible in mature appressoria, after migration of the nucleus into the appressorium.

Text modification, line 199-203:

Confocal laser scanning microscopy detected GFP fluorescence specifically in the nucleus of mature appressoria, but not in conidia, nor germinating hyphae and young appressoria (**Fig 5D).** These experiments showed that BIP1 is an appressorium-specific TF with a nuclear localization, expressed after the migration of the nucleus into the appressorium.

3- Figure 6 and 7. I’m curious as to how many times the DNA-binding assays were performed? If multiple replicates already exist, it might be nice to quantify these.

**Answer**: DNA-binding assays were performed at least as duplicates, and for some samples over 5 times.

4- A very minor point: Lines 360 and 408 mention “randomly” selecting genes/promotors to study further, but I couldn’t see any mention in the methods of how this process was randomized. Did the authors just select their favorites or was this truly randomized, and if so how?

**Answer**: The selection for qPCR was only partly random, as we selected at random genes from some functional categories. The text has been modified accordingly. The selection of random promoters for binding assay search is likely not relevant. It has been replaced by a whole genome analysis of promoters.

Text modification, lines 229-230:

qRT-PCR experiments were performed with eight BIP1-regulated genes from three functional categories…

Text modification, lines 252-258:

Analysis of promoter sequences 1 kb upstream from the start codon, showed an enrichment of a conserved TGACTC sequence similar to the GCN4-like binding motif in the promoters of the 40 genes down-regulated in *Δbip1* appressoria. Indeed, using MEME, the motif was found in 36% of the BIP1-regulated genes and only 12% of the 12.593 promoters of *M. oryzae* (FIMO analysis, p-value 10^-6^, [46]). Searching for more degenerated versions of the motif using an in-house script, identified the motif in one or multiple copies in the promoters of all 40 genes.

**Part II – Major Issues: Key Experiments Required for Acceptance**

**Reviewer #1**:

1. Deletion of BIP gene still formed well-melanized appressoria, but did not penetrate, even wounded leaves. Instead of just saying defective, authors may check the turgor of appressoria formed by deletion mutant using plasmolysis/cytolysis. Further it would be better authors present all phenotypes of the mutant measured in this study including stress responses to speculate more on defective in invasive growth.

**Answer**: We have assayed appressorium turgor using a range of PEG concentrations. These additional experiments have shown that the Δ*bip1* mutant was similar to wild-type for appressorium turgor (S2 Fig). Additional experiments have also shown that the *Δbip1* mutant was similar to wild-type for sensitivity to stress (cell wall, oxidative, S5 Fig).

Text modification, lines 145-150:

The *MGG_08118* deletion mutant, differentiated melanized appressoria that were indistinguishable from WT ones in shape, number and turgor (**Fig 3**, **S2 Fig**). Therefore, the loss of *MGG_08118* did not impair the differentiation, turgor build-up and maturation of appressoria. *MGG_08118* deletion mutants were also not affected in their conidiation (**S3 Fig**), mycelial growth (**S4 Fig**), or resistance to cell wall and oxidative stress (**S5 Fig**).

2. Expression of BIP1 gene.

2.1- There is difference between on the Teflon (conidia and appressoria) and barley leaves. What is the explanation, plant (e.g. how about on the onion?) or host effect?

**Answer**: There is no difference in the level of expression of *BIP1* in appressoria differentiated on Teflon or barley leaves (almost the same level as shown in Figure 5A and 5B). *BIP1* mRNA was indeed detected in isolated conidia. However, the lack of detection of BIP1 mRNA by qRT-PCR at a very early stage of infection (only conidia on plant leaves, data not shown), is likely due to the low fungal biomass of these samples. Indeed, these samples (0 hpi, 4 hpi) were not included in Figure 5, as the expression of constitutive fungal genes was not detected. We have used infected leaves samples from 8 to 72 hpi for Figure 5B, as the expression of constitutive fungal genes was detected.

2.2- RNAseq was done the samples from Teflon. However, it is not clear what type of RNA sample used for oligonucleotide microarray. Authors identified 42 down regulated genes from bip1 deletion mutant and binding motif TGACTCG. These 42 were from microarray data. How about RNAseq data? Since authors already has RNAseq data of bip1 deletion mutant during appressorium formation, more comprehensive genome-wide identification/analysis would be available.

**Answer**: Oligonucleotide microarray experiments were performed with RNA extracted from appressoria differentiated on Teflon (cf methods). Published RNAseq experiments from N. Talbot’s lab (Osés-Ruiz et al., 2021) were used as an external reference for appressorium-expressed genes in this study (Table 1). We do not have RNAseq for *Δbip1* appressoria. New RNAseq experiments go beyond the scope of the study.

3. Identification of BIP1 binding motif. It would be better if authors could provide genome-wide presence of this motif, not limited to 42 genes!

**Answer**: We have performed a MEME (FIMO) analysis of all the promoters of *M. oryzae* for the occurrence of the GCN4/bZIP-binding DNA motif (TGACTC) to compare to the occurrence in BIP1 target genes.

Text modification, lines 252-258:

Analysis of promoter sequences 1 kb upstream from the start codon, showed an enrichment of a conserved TGACTC sequence similar to the GCN4-like binding motif in the promoters of the 40 genes down-regulated in *Δbip1* appressoria. Indeed, using MEME, the motif was found in 36% of the BIP1-regulated genes and only 12% of the 12.593 promoters of *M. oryzae* (FIMO analysis, p-value 10^-6^, [46]). Searching for more degenerated versions of the motif using an in-house script, identified the motif in one or multiple copies in the promoters of all 40 genes.

**Reviewer#2**: Comments:
1- Many experiments conducted in this manuscript are performed in barley. All experiments should be conducted using rice plants to fully validate the results obtained using barley. This is especially important when conducting gene expression profile and microscopy analyses.

**Answer**: We have performed additional pathogenicity experiments using rice, including the analysis of plant penetration by confocal microscopy (S3 Fig). In addition, we have added qRT-PCR analysis of the expression of 15 BIP1-regulated genes during early rice infection in wild-type and *Δbip1* (S2-Table).

Text modification, lines 151-154:

However, the ability of this mutant to penetrate into barley and rice epidermal cells was completely abolished (**Fig3**, **S6 Fig**). Indeed, confocal microscopy showed that the mutant was arrested before the development of early infectious hyphae, and, presumably, did not produce a penetration peg (**Fig3, S6 Fig**).

Text modification Lines 243-245:

Analysis of the expression of 15 BIP1-regulated genes during initial stages of rice infection (16 hai) showed that the induction of six of them was completely abolished and five others were strongly downregulated (S2 Table).

2- Lines 150-151: Referencing Figure 1 and Figure 3 does not support the claims by the authors. Results from infection on wounded leaves must be shown in the manuscript and should be performed using rice.

**Answer**: We have performed additional pathogenicity experiments using wounded rice and barley leaves that are provided in S7 Figure.

Text modification, lines 154-156:

The *MGG_08118* deletion mutant was also unable to infect wounded leaves indicating that the formation of invasive infectious growth was also impaired (**S7 Fig**).

3- In line 223, the authors mention BIP1 has a nuclear localization signal. However, this is not represented as mentioned in Supplementary Figure 2. It would be ideal if the authors included a schematic representation of the sequence of BIP1, highlighting the NLS and other domains within the gene.

**Answer**: We have added an illustration that shows the domains of BIP1 in the context of the full-length protein as a supplementary Figure 8.

Text modification, Lines 161-163:

The protein domain search tools CD (CD14688), Panther (PTHR11462), and Superfamily (SSF57959), identified a bZIP domain in BIP1 protein sequence (**S8B Fig**), but not Pfam.

4- In addition to the previous point, the authors can generate truncations of the BIP1 protein, with or without the NLS, to show the functionality of the NLS in BIP1. This would strongly support the findings from the authors in Figure 5D that indeed BIP1 localizes to the nucleus of the appressoria via its operational NLS.

**Answer**: We think that experiments that address the function of individual domains and motifs of BIP1, are beyond scope of this study. They are interesting, but not directly linked to the numerous new and original findings of our study.

5- In the materials and methods on line 784, the authors state that they use onion epidermis for experiments. However, there are no figures depicting the results of using onion for microscopy work. The authors should include the results from these experiments that are listed in this section.

**Answer**: Onion epidermis experiments were not used in this study and they have been removed from methods section.

6- While the authors state that there is no additional phenotype seen in this BIP1 mutant, the authors need to include morphological characterization in axenic conditions, conidiation data, and appressorium formation assays using the BIP1 mutant and WT strain.

**Answer**: We have performed new experiments to compare the *Δbip1* mutant, the complemented strain and the wild type isolate for growth in axenic culture, conidiation, and appressorium differentiation. The corresponding data are in S2 Figure, S3 Figure, S4 Figure and S5 Figure. They confirm that the *Δbip1* mutant is indistinguishable from the wild-type for mycelial growth, conidiation and appressorium differentiation.

6- This manuscript would benefit from conducting cell wall integrity and oxidative stress tests on the BIP1 mutant strain compared to the WT to see if there are additional defects with the mutant strain. Even if these findings show negative data, the authors can include these results in the supplementary section to depict the full profile of this fungal mutant strain.

Answer: We have added results from experiments that compare *Δbip1* and wild type for their sensitivity to cell wall integrity and oxidative stress. We provide data and Figures corresponding these experiments as supplementary Figures (S5 Fig). They show that the *Δbip1* mutant and the wild-type have similar sensitivity to cell wall and oxidative stresses.

Text modification, lines 148-150.

*MGG_08118* deletion mutants were also not affected in their conidiation (**S3 Fig**), mycelial growth (**S4 Fig**), or resistance to cell wall and oxidative stress (**S5 Fig**).

7- How can the authors guarantee that the strain is not penetrating rather than not proliferating inside the cell? Figure 1A shows small lesions when infecting barley with the BIP1 mutant. If this mutant is highly involved in penetration peg formation, the authors should present penetration rate statistics on rice between the WT and BIP1 mutant strain to show the overall percentage of infection. Additional experiments need to be conducted to show that the penetration peg is defective in the BIP1 mutant. Microscopy work using rice and the BIP1 mutant strain at varying time points of infection would allow readers to see defects in penetration peg formation and invasive hyphae growth.

**Answer**: We have performed additional confocal microscopy analysis of *Δbip1* mutant infection attempts on barley and rice leaves to complete the transmission microscopy analyses that were in the initial submission. These novel experiments clearly discriminated the fungal cells on the surface of the leaves (conidia, hyphae, appressoria) from infection structures such as infectious hyphae developing inside epidermal cells. Using this approach, we did not detect infective hyphae in the mutant, even not very small, early infective hyphae such as those formed in the *M. oryzae* tig1 histone deacetylase mutant (Ding et al. 2010, figure 3 & 4; doi: 10.1105/tpc.110.074302) (Figure 3 B and Supplemental Figure 3). Confocal microscopy also did not show any sign for the formation of a penetration peg. Since early penetration pegs may be difficult to visualize by confocal microscopy, we remained cautious in the interpretation of this result

Text modification, lines 151-154:

However, the ability of this mutant to penetrate into barley and rice epidermal cells was completely abolished (**Fig3**, **S6 Fig**). Indeed, confocal microscopy showed that the mutant was arrested before the development of early infectious hyphae, and, presumably, did not produce a penetration peg (**Fig3, S6 Fig**).

8- How is the BIP1 localization in the conidia versus the mycelia in the 3xGFP strain? This data would complement the gene expression profile presented in Figure 5A.

**Answer**:

The BIP1-3xeGFP strain shows no fluorescence in conidia, germinating hyphae, or young appressoria and we conclude that the BIP1-3xeGFP protein is not expressed under these conditions

9- Authors should consider statistics analysis for the pathogenicity assays and appressoria formation.

**Answer**: The *Δbip1* mutant is completely non-pathogenic on rice leaves and shows no disease symptoms in spray or spot inoculation tests. We therefore think that it is not useful to provide quantitative data for rice pathogenicity assays. Quantitative analysis of appressorium formation has been added as Figure 3 A.

Reviewer #3: n/a

**Part III – Minor Issues: Editorial and Data Presentation Modifications**
Please use this section for editorial suggestions as well as relatively minor modifications of existing data that would enhance clarity.

Reviewer #1:

1. Promotor mutation of MGG08381. Are there any phenotypes including appressorium formation and invasive growth?

Answer: The deletion mutant of *MGG08381* was not obtained. Therefore, we cannot answer to this question.

2. In Figure 5, what are the units of expression levels (A and B)? Sp should be Conidia?

Answer: Sp has been modified to conidia. Units of expression levels are expression levels relative to the constitutive gene *ILV5*. This information is provided in the caption of Figure 5.
