# Supplementary material for: The bZIP transcription factor BIP1 of the rice blast fungus is essential for infection and regulates a specific set of appressorium genes
Source: PLoS Pathog. 2024 Jan 22;20(1):e1011945. doi: 10.1371/journal.ppat.1011945 (PMC10833574; doi:10.1371/journal.ppat.1011945)
Supplement: Attachment — Submitted filename: reponse_editeur_final.docx [file ppat.1011945.s017.docx]

Dear Editor,

We would like to thank you for your comments, which helped us to improve our manuscript and to clarify an important aspect of the *Δbip1* mutant phenotype. Below, in the decision letter, we provide a detailed answer to your requests.

Sincerely

Karine Lambou

**Response:**

As requested, we performed standard penetration assays on cellophane membranes (NC2380, Thermofisher), and found that the *Δbip1* mutant was indistinguishable from the wild-type and the complemented strain in these experiments. Like the wild-type strain, the *Δbip1* mutant penetrated through appressoria into the cellophane membrane, where it differentiated bulbous pseudo-infectious hyphae growing within the membrane under the appressoria. This phenotype shows that BIP1 is not required for the differentiation of the penetration peg or primary or infectious hyphae and strengthens the hypothesis that BIP1 is crucial for the early establishment of the fungus within plant cells.

To integrate these new results, we have changed the manuscript in the following way:

**Addition of photos documenting the cellophane growth assays as Figure 4** and **modification of the corresponding legend** by addition of the following text:

Lines 1186-1190:

Fig 4. Appressorium-mediated penetration of *Δbip1* into cellophane membranes.

Conidia of wild-type (P1.2), *Δbip1* mutant and *Δbip1* complemented strains were deposited on cellophane membrane, and appressoria (black arrows) and pseudo-infection hyphae growing into the membrane (white arrows) were visualized by differential interference contrast microscopy at 48 hai and 72 hai. Bar=10μm.

**Modification of the abstract :**

-Lines 38-42:

BIP1 is essential for appressorium-mediated penetration into plant leaves but not into artificial cellophane membranes. This phenotype suggests that BIP1 is not implicated in the differentiation of the penetration peg but is necessary for the initial establishment of the fungus within plant cells.

-Lines 52-54:

Overall, our results show that BIP1 coordinates the expression of pathogenicity-related genes during the initial establishment of the fungus within host leaves.

**Modification of the Introduction:**

Lines 122-124:

*BIP1* deletion mutants were non-pathogenic on rice and barley, and differentiated melanized appressoria unable to penetrate into host leaves but penetrated into artificial cellophane membranes.

**Addition of the following text to the Results:**

Lines 158-169:

The *MGG_08118* deletion mutant was also unable to infect wounded leaves indicating that the formation of invasive infection hyphae was also impaired in planta (**S7 Fig**). In contrast, the *MGG_08118* deletion mutant was able to penetrate through appressoria into artificial cellophane membranes. As wild-type and the complemented strain, the *MGG_08118* mutant differentiated bulbous pseudo-infection hyphae within the membrane under the appressoria, forming a star-like network of hyphae within the cellophane (**Fig4**). These findings indicated that *MGG_08118* is not involved in the differentiation of the penetration peg, or primary infection hyphae. Instead, they suggested that *MGG_08118* is essential for the early establishment of the fungus in its host plant, as evidenced by the absence of infection hyphae in the epidermal plant cell below the appressoria (**Fig3**, **S6 Fig**).

**Addition of the following text to the Discussion**:

-Lines 337-375:

*M. oryzae* mutants unable to infect wounded plant as *Δbip1,* were affected in genes essential for penetration peg formation like *PLS1, NOX2, MPS1* or *MST12* [15,42,47,49,50], or crucial for establishing primary infection hyphae within infected plant cells [51]. Cellophane penetration assays conducted with the *Δbip1* mutant suggested that *BIP1* is not directly involved in the differentiation of the penetration peg. Instead, it is more likely that BIP1 is essential for the early establishment of the fungus within plant cells. Indeed, as the wild type, the *Δbip1* mutant penetrated through appressoria into artificial cellophane membranes, where it formed bulbous pseudo-infection hyphae growing within the membrane under the appressoria (**Fig4**).

In most other *M. oryzae* mutants with defects in early invasive infectious growth, primary infection hyphae (PIH) are formed in the first infected cell before being arrested [52]. However, a few mutants exhibited a phenotype similar to *Δbip1,* characterized by either the absence or the presence of a very short PIH. Examples of this second type included deletion mutants of the histone deacetylase *TIG1,* the transcription factor *ATF1* or the iron metabolism protein *DES1* [51,53,54]. These three mutants differentiate a few short PIH that are rapidly blocked in the first infected epidermal cell. These mutants displayed an increased sensitivity to H_2_0_2_, which was not observed for *Δbip1.* Their attempted infection triggers strong plant defenses in the first infected cell including increased H_2_0_2_ production, potentially accounting for their arrest. In the case of *Δatf1 and Δdes1,* pathogenicity could be restored by pharmacological inhibition of ROS production, further supporting that non-pathogenic phenotypes are primarily due to an inability to overcome plant defenses. Some *M. oryzae* mutants displayed phenotypes more similar to *Δbip1.* For instance, the deletion mutant of the MIG1 transcription factor from the MPS1/SLT2 signaling pathway was unable to form PIH within rice and onion cells, but like *Δbip1* could penetrate cellophane. Additionally, it invaded heat-killed rice and onion leaf epidermal cells [55]. The deletion mutant of the transcription factor WOR1/GTI1 formed very short PIH within rice and onion cells [56]. Preliminary characterization of its regulatory network suggested differences from that of BIP1 (see below). Based on these examples from the literature, we conclude that BIP1 is essential for early invasive infectious growth. This hypothesis may be further examined in future studies by elucidating its role in establishing within the first infected cell and/or overcoming plant defenses.

-Lines 501-507:

For the previously mentioned transcription factor WOR1, whose mutant, like *Δbip1*, is unable to invade the first infected cell, only selected genes were analyzed for altered expression in mutant appressoria. The effector *BAS2,* which was downregulated in *Δbip1,* was overexpressed in *Δwor1.* On the reverse, the effectors *BAS1*, *BAS4*, *AVR-Pita*, *Pwl2* and *MC69*, whose expression were not altered in *Δbip1,* were down-regulated in the WOR1 mutant. Therefore, we assume that the regulatory networks of BIP1 and WOR1 are distinct.

**Addition of text to the Material and methods**:

Lines 591-596:

Penetration assays on artificial membranes were performed by depositing droplets of conidia (5.10^4^ conidia.mL^-1^) containing 10 µM hexadecanediol on cellophane membrane (NC2380, thermofisher) previously placed on the top of a 3% (w/v) water–agar layer. The cellophane membranes were observed 48 and 72 hours after inoculation of conidial suspensions by differential interference contrast microscopy (Nikon Eclipse Ni) and z-stacks were realised.

**Addition of the five following references to the references list:**

Lines 957-972:

52.	Tan J, Zhao H, Li J, Gong Y, Li X. The Devastating Rice Blast Airborne Pathogen *Magnaporthe oryzae*—A Review on Genes Studied with Mutant Analysis. Pathogens. 2023;12: 379. doi:10.3390/pathogens12030379

53.	Guo M, Guo W, Chen Y, Dong S, Zhang X, Zhang H, *et al.* The Basic Leucine Zipper Transcription Factor Moatf1 Mediates Oxidative Stress Responses and Is Necessary for Full Virulence of the Rice Blast Fungus *Magnaporthe oryzae*. MPMI. 2010;23: 1053–1068. doi:10.1094/MPMI-23-8-1053

54.	Sarkar A, Roy-Barman S. Spray-Induced Silencing of Pathogenicity Gene *MoDES1 via* Exogenous Double-Stranded RNA Can Confer Partial Resistance Against Fungal Blast in Rice. Front Plant Sci. 2021;12: 733129. doi:10.3389/fpls.2021.733129

55.	Mehrabi R, Ding S, Xu J-R. MADS-Box Transcription Factor Mig1 Is Required for Infectious Growth in *Magnaporthe grisea*. Eukaryot Cell. 2008;7: 791–799. doi:10.1128/EC.00009-08

56.	Li Y, Wang G, Xu J-R, Jiang C. Penetration Peg Formation and Invasive Hyphae Development Require Stage-Specific Activation of *MoGTI1* in *Magnaporthe oryzae*. MPMI. 2016;29: 36–45. doi:10.1094/MPMI-06-15-0142-R
