# Supplementary material for: The bZIP transcription factor BIP1 of the rice blast fungus is essential for infection and regulates a specific set of appressorium genes
Source: PLoS Pathog. 2024 Jan 22;20(1):e1011945. doi: 10.1371/journal.ppat.1011945 (PMC10833574; doi:10.1371/journal.ppat.1011945)
Supplement: Attachment — Submitted filename: 2023_12_26_reponse_editeur.docx [file ppat.1011945.s018.docx]

Dear Editor,

Thank you for your comments, which helped to further improve our manuscript. Below, we provide a detailed answer to the three points you raised in your decision letter.

Sincerely

Karine Lambou

1. Bip1 is shown to not be required for penetration peg formation or, according to the cellophane assay, penetration, but is required for establishing IH in the first infected cell. This is stated in the discussion, but in the abstract and elsewhere, there are statements such as "BIP1 is essential for appressorium-mediated penetration into plant leaves but not into artificial cellophane membranes", which is misleading, as it suggests a role for Bip1 in penetration on leaves. Please make clear throughout that on the basis of the results, Bip1 is not likely required for penetration but is required for establishing early biotrophic growth in host cells.

We perfectly agree, and have removed the inconsistencies that remained in the abstract and the discussion.

**Modification of the abstract :**

Lines 37-39

“BIP1 is required for the infection of plant leaves, even if they are wounded, but not for appressorium-mediated penetration of artificial cellophane membranes.”

Lines 48-50:

“Interestingly, this BIP1 network is different from other known infection-related regulatory networks, highlighting the complexity of gene expression control during plant-fungal interactions.”

Lines 54-62:

“In summary, our findings demonstrate that BIP1 is critical for the expression of early invasion-related genes in appressoria. These genes are likely needed for biotrophic invasion of the first infected host cell, but not for the penetration process itself. Through these mechanisms, the blast fungus strategically anticipates the host plant environment and responses during appressorium-mediated penetration.”

**Modification of the summary author :**

Lines 69-83 :

“Here, we have identified in a pathogenicity mutant screen using random insertional mutagenesis, the novel M. oryzae bZIP transcription factor BIP1 that is essential for the infection. BIP1 is not implicated in the development of appressoria or the subsequent penetration of host leaves, but is necessary for the initial establishment of the fungus within plant cells. BIP1 orchestrates the expression of a unique set of early invasion-related genes within appressoria, encoding secreted effectors, enzymes, secondary metabolism-related enzymes, and signaling membrane receptors. Our experimental data suggest that BIP1 controls their expression by interacting directly with a TGACTC motif present in their promoters. Remarkably distinct from other known pathogenicity networks, the BIP1 regulatory network underscores the intricate control of fungal gene expression during infection. BIP1 seems to prepare *M. oryzae* for early biotrophic growth during appressorium-mediated penetration.”

**Modification of the conclusion**

Lines 503-505 :

“BIP1 is required for the expression of a unique set of genes essential for early invasive infectious growth.”

Lines 519-523:

“BIP1 stands out for its distinctive role in regulating the expression of a specific set of early invasion-related genes within the appressorium. This unique function equips the fungus, even before appressorium-mediated penetration, to adapt to the plant's cellular environment and anticipate its defense responses.”

2. Impaired penetration is shown for barley (Fig 3) and rice (S6 Fig). Please note that the figure legend for S6 Fig has been swapped with that for S4Fig, please correct.

We have corrected the wrong numbering of S Fig legends (lines 1073-1107).

Also, many readers are interested in rice blast rather than barley blast. Therefore, it is important that the confocal images in S6Fig, stained with WGA, be moved to the main figure alongside the barley results.

We have included S6Fig into Figure 3 as an additional panel (panel C), lines1179:1188.

However, these image panels need to be improved. Both a DIC or brightfield panel and a merged panel should be included, and the appressoria on the surface and points of IH spread to neighbouring cells should be indicted.

We now show the overlay images in the Figure 3 and separate bright field, fluorescence and merged images in S6 Fig. In addition, we have labelled conidia, appressoria, and invasive hyphae on the pictures.

The current images are hard to interpret and it looks to me like the stained IH for WT and Bip1 complement are hyphae on the surface of the leaf. It may be necessary to show surface images of the three strains to indicate appressoria, and a lower depth image to show IH produced by the WT and the complement.

P1.2 wild-type displays typical invasive hyphae inside plant cells, and we have labelled them accordingly in Figure 3 and S6 Fig. For the complemented mutant, appressoria and conidia are absent from the pictures we have, and we have therefore removed it from the figure. We think results from this line are not essential for this figure.

We would like to use actual panel C with a complete Z-stack, without modifications or supplemental images, to avoid too complex presentation of data. However, if you consider this an important issue, we could also add, as supplementary material in S6 Fig, two separate z-stacks for the wild-type one containing the leaf surface, appressoria and conidia and one of deeper sections with only infectious hyphae. Or, alternatively, show a 3D projection similar to the one in panel B.

3. Because bip1 can penetrate cellophane but cannot infect wounded tissues, the most parsimonious explanation is that Bip1 is required for expressing genes in appressoria that are needed for establishing biotrophic growth, but not for penetration. This important point needs to stressed throughout, as it suggests genes required by IH for colonizing hot cells are "pre-expressed" before penetration, perhaps in order to condition the fungus for the host cell prior to infection. I do not believe this was clearly stated in the text, but it is a main finding of the work and perhaps should be added to the abstract.

We perfectly agree and have emphasized this idea at the end of the abstract and the conclusion paragraph of the discussion

**Modification of the abstract:**

Lines 53-62:

“In summary, our findings demonstrate that BIP1 is critical for the expression of early invasion-related genes in appressoria. These genes are likely needed for biotrophic invasion of the first infected host cell, but not for the penetration process itself. Through these mechanisms, the blast fungus strategically anticipates the host plant environment and responses during appressorium-mediated penetration.”

**Modification of the summary author :**

Lines 69-83 :

“Here, we have identified in a pathogenicity mutant screen using random insertional mutagenesis, the novel M. oryzae bZIP transcription factor BIP1 that is essential for the infection. BIP1 is not implicated in the development of appressoria or the subsequent penetration of host leaves, but is necessary for the initial establishment of the fungus within plant cells. BIP1 orchestrates the expression of a unique set of early invasion-related genes within appressoria, encoding secreted effectors, enzymes, secondary metabolism-related enzymes, and signaling membrane receptors. Our experimental data suggest that BIP1 controls their expression by interacting directly with a TGACTC motif present in their promoters. Remarkably distinct from other known pathogenicity networks, the BIP1 regulatory network underscores the intricate control of fungal gene expression during infection. BIP1 seems to prepare *M. oryzae* for early biotrophic growth during appressorium-mediated penetration.”

**Modification of the conclusion:**

Lines 503-505:

“BIP1 is required for the expression of a unique set of genes essential for early invasive infectious growth.”

Lines 519-523:

“BIP1 stands out for its distinctive role in regulating the expression of a specific set of early invasion-related genes within the appressorium. This unique function equips the fungus, even before appressorium-mediated penetration, to adapt to the plant's cellular environment and anticipate its defense responses.”
